# Associations of sleep quality and fall risk among older adult outpatients at Thai Binh Medical University Hospital, Northern Vietnam

**DOI:** 10.3389/frsle.2024.1486794

**Published:** 2025-01-23

**Authors:** Nguyen The Diep, Doan Anh Tuan, Tien Van Nguyen, Hoang Minh Anh, Dao Huy Cu

**Affiliations:** ^1^Department of Traumatology, Thai Binh University of Medicine and Pharmacy, Thai Binh, Vietnam; ^2^Department of Traumatology, Dong Anh General Hospital, Hanoi, Vietnam; ^3^Faculty of Public Health, Thai Binh University of Medicine and Pharmacy, Thai Binh, Vietnam; ^4^Center for Population Health Sciences, Hanoi University of Public Health, Hanoi, Vietnam; ^5^Technical Department, Vietnam Center for Community Health and Injury Prevention, Hanoi, Vietnam

**Keywords:** accidental falls, aged, sleep wake disorders, sleep quality, insomnia, Northern Vietnam

## Abstract

**Introduction:**

Poor sleep quality negatively impact health, reducing quality of life and increasing disease risk. For the elderly, poor sleep quality lead to fatigue and reduced mobility, increasing the risk of falls. In Vietnam, no studies have explored the relationship between sleep quality and fall risk in the elderly. This study aimed to determine the correlation between sleep quality and fall risk among the elderly, providing knowledge for fall prevention in this population in Vietnam.

**Methods:**

A cross-sectional study was conducted on 404 elderly patients who visited and were treated as outpatients at Thai Binh University Hospital from October 2023 to June 2024. Direct interviews were conducted using the Fall Risk Questionnaire (FRQ) from STEADI-CDC-2017 (USA), and the Pittsburgh Sleep Quality Index (PSQI) with a cut-off score of 7 to assess sleep quality. The FRQ tool used a cut-off score of 4 to evaluate fall risk.

**Results:**

The percentage of elderly at risk of falls was 19.6%. The average PSQI score was 11.0 ± 2.7, with 358 (88.6%) elderly patients showing poor sleep quality. Logistic regression analysis showed that higher PSQI scores in the elderly significantly correlated with an increased risk of falls (OR = 1.16, 95% CI: 1.02–1.32, *p* = 0.03). Elderly individuals with the highest PSQI scores had a higher risk of falls compared to those with the lowest PSQI scores.

**Conclusion:**

Poor sleep quality are closely related to fall risk among the elderly, alongside other factors such as religion, education, comorbidities, and participation in recreational activities.

## 1 Introduction

Falls are the leading cause of injury-related death and rank as the seventh leading cause of death among the elderly (Elderly) (Burns and Kakara, 2018). In the United States, according to reports from the Centers for Disease Control and Prevention (CDC), ~14 million elderly individuals (28%) fall each year out of a total of 36 million falls (Centers for Disease Control and Prevention, 2023a). It is estimated that about 8 million fall-related injuries occur annually, with ~37% of those who fall reporting injuries requiring medical treatment or temporary mobility restrictions (Moreland et al., 2020). According to reports by World Health Organization (WHO), an estimated 1.5–1.9 million elderly individuals fall annually in Vietnam, with 5% of these cases requiring hospitalization due to injuries (World Health Organization, 2008). A study by Nguyen et al. (2024) showed that about 18.3% of injuries from falls occur annually, and the fall rate among the elderly with knee osteoarthritis is 23.3% (Cao and Le, 2023). On the other hand, a study by Tang et al. (2022) found that the risk of falls among the elderly living in rural areas is high (47.8%). However, these studies generally assess fall status and fall risk among the elderly living in the community.

The risk of falls among the elderly has been shown to be associated with various factors, including both subjective and objective factors. Moreover, recent reports have demonstrated a correlation between fall risk and sleep quality in the elderly (Kuo et al., 2010; Stone et al., 2014). It is clear that poor sleep quality, particularly among the elderly with sleep disorders, increases the risk of falls due to its impact on balance and cognitive function (Gu et al., 2010). However, the conclusions drawn from these studies remain controversial. Furthermore, these studies assess sleep duration and quality using inconsistent methods and do not fully reflect individual sleep parameters. Therefore, the relationship between sleep quality and fall risk in the elderly has not yet been clarified.

A sleep quality assessment tool that has recently been widely and effectively applied is the Pittsburgh Sleep Quality Index (PSQI). The PSQI is a questionnaire consisting of 18 items that assess sleep quality based on seven different aspects (Buysse et al., 1989). Additionally, the fall risk assessment tool has been proven to reduce falls and fall-related injuries among the elderly in the community (Tang et al., 2022). A study on elderly men found a correlation between sleep quality (measured by the PSQI) and fall risk (Stone et al., 2014). Understanding the relationship between sleep quality and fall risk in the elderly can help identify high-risk groups in the community, thereby proposing effective prevention and intervention measures. For this reason, we conducted this study with the objective of evaluating the correlation between fall risk and sleep quality in elderly outpatients at Thai Binh University Hospital.

## 2 Materials and methods

### 2.1 Subjects, location, and research duration

The study was conducted through a cross-sectional survey of elderly individuals (aged ≥60 years) living in Thai Binh province. The elderly were selected for the study if they met the following criteria: aged ≥60 years, visited and received outpatient treatment at the Department of Examination, Thai Binh University Hospital from October 2023 to June 2024; had permanent residency and had been living in the study area for at least 12 months; and voluntarily agreed to participate in the study. The study did not include elderly individuals with impaired consciousness or coma, those with previously diagnosed and currently treated psychotic disorders, those with hearing or visual impairments, physical deformities, or those who did not reside regularly in the study area.

We applied the formula for estimating a proportion to determine the sample size for the study:


$$\begin{array}{l} n=Z^{2}+\left(1-\frac{\alpha }{2}\right)\frac{p\left(1-p\right)}{d^{2}} \end{array}$$

In which, *n*: the total number of subjects required for the study; *Z*: the confidence coefficient calculated based on α, where α = 0.05 with a 95% confidence interval [*Z*_(1−α/2)_ = 1.96]; *d*: the desired margin of error, set at *d* = 0.05; *p*: the proportion of elderly individuals at high risk of falls. According to the study by Tang Thi Hao, this proportion is 47.8%, so p is set at 0.478 (Tang et al., 2022). Substituting into the formula, n is calculated to be 384. In practice, the research team surveyed 404 elderly individuals who met the inclusion criteria for the study.

### 2.2 Sampling method

In the current study, we used a convenience sampling method to select 404 elderly patients who met the inclusion criteria for participation in the study.

### 2.3 Data collection tools and methods

The questionnaire was designed based on predefined variables. A pilot survey was conducted with 20 elderly individuals at Thai Binh University Hospital to evaluate the content and presentation of the questionnaire. We conducted direct interviews with research participants using the pre-prepared questionnaire. The interviewers were experienced in community surveys and were fully trained on the tools and methods of data collection.

### 2.4 Evaluation criteria

#### 2.4.1 Fall risk among the elderly

The Fall Risk Questionnaire (FRQ) from the STEADI-CDC-2017 (USA), was used to assess fall risk among the elderly (Centers for Disease Control Prevention, 2023b; Stevens and Phelan, 2013). The questionnaire consists of 12 questions with a total score of 14 (Questions C1 and C2 have two score levels: 2 and 0), summing up the scores for each “Yes” answer. The scale has good reliability, validity, and high sensitivity in fall risk assessment among the elderly (Kitcharanant et al., 2020; Song et al., 2020). The elderly were classified as having a fall risk if their total score was 4 or higher and as not at risk if their total score was below 4.

#### 2.4.2 Sleep quality among the elderly

The sleep quality of the elderly was assessed using the Pittsburgh Sleep Quality Index (PSQI) questionnaire, which consists of 18 questions covering seven aspects: subjective sleep quality, sleep latency, habitual sleep efficiency, sleep disturbances, use of sleeping medications, and daytime dysfunction. The elderly were considered to have poor sleep quality if their total PSQI score was >7, and good sleep quality if the total PSQI score was 7 or less (Buysse et al., 1989; Carpenter and Andrykowski, 1998; Graco et al., 2023; Gomes et al., 2018).

### 2.5 Data processing and analysis

The collected data were entered twice using EpiData 3.0 software. Data cleaning: after data entry was completed, the data were cleaned by comparing the two entries and correcting any errors during data entry. The data were presented as counts and percentages (%) for categorical variables and as means and standard deviations for continuous variables. Factors related to fall risk and the correlation between fall risk and sleep quality were determined using logistic regression models.

Variables were screened by examining the unadjusted relationship between each risk factor and the outcome using logistic regression. We assessed the relationship between the tertiles and quartiles of PSQI scores and fall risk in the elderly using logistic regression analysis. The first tertile and quartile were used as references to estimate odds ratios (OR) and 95% confidence intervals (CI). In the first model, we adjusted for age and gender. In the second model, we additionally adjusted for any falls in the previous year. In the third model, we further adjusted for education level, smoking history, alcohol consumption history, participation in leisure activities, and comorbidities.

All analyses were conducted using SPSS 22.0 software to correctly account for complex survey sampling structures, including stratification, clustering, and unequal weighting. The relationship between dependent variables and fall risk in the final model was reported as adjusted odds ratios with 95% confidence intervals (95% CI) and P-values.

### 2.6 Research ethics

The study was approved by the Ethics Committee of Thai Binh University of Medicine and Pharmacy under Decision No. 0224/IRB dated July 22, 2024. The research participants were informed about the purpose and content of the study before answering the questions and were entirely free to refuse to participate if they chose. Participation in the study was entirely voluntary, and all information about the research subjects was kept confidential. All information from the research participants was encoded and secured. The data and information collected in the reports were committed to being used solely for research purposes and not for any other purposes. The research results and proposed recommendations will be used to improve health and healthcare services for patients visiting and receiving treatment at Thai Binh University Hospital.

## 3 Results

### 3.1 General characteristics of the study subjects

The distribution of general characteristics of the study subjects according to fall risk shows that female elderly accounted for 61.1%, as shown in Table 1. The age group 60–70 had the highest proportion (58.4%). The predominant education level was junior high school, with 48.0%, and the comorbidity rate was 50.2%. Most elderly participants in the study had no previous history of falls (83.7%) and regularly engaged in physical exercise (86.8%) as well as recreational activities (95.8%). The proportion of elderly with good sleep quality was only 11.4%, while poor sleep quality accounted for 88.6%. The differences in fall risk rates among elderly groups based on age, education level, religion, history of falls, comorbidities, and participation in recreational activities were statistically significant (*p* < 0.05).

**Table 1:** Characteristics of the study subjects according to fall risk^*^.

**Variables**	**Total (*n* = 404)**	**Fall risk**		***P*-value^a^**
		**No (*****n*** = **325)**	**Yes (*****n*** = **79)**	
**Gender**				
Male	157 (38.9)	132 (40.6)	25 (31.6)	0.14
Female	247 (61.1)	193 (59.4)	54 (68.4)	
**Age (years)**				
60–70	236 (58.4)	200 (61.5)	36 (45.6)	**0.02**
70–80	132 (32.7)	101 (31.1)	31 (39.2)	
≥80	36 (8.9)	24 (7.4)	12 (15.2)	
**Religion**				
No	377 (93.3)	312 (96.0)	65 (82.3)	**<0.001**
Yes	27 (6.7)	13 (4.0)	14 (17.7)	
**BMI (kg/m** ^2^ **)**				
Normal weight	246 (60.9)	198 (60.9)	48 (60.8)	0.88
Overweight	103 (25.5)	84 (25.8)	19 (24.1)	
Obesity	55 (13.6)	43 (13.2)	12 (15.2)	
**Education**				
Primary school and below	54 (13.4)	39 (12.0)	15 (19.0)	**0.01**
Junior school	194 (48.0)	166 (51.1)	28 (35.4)	
High school	104 (25.7)	75 (23.1)	29 (36.7)	
College and above	52 (12.9)	45 (13.8)	7 (8.9)	
**Smoking history**				
No	353 (87.4)	287 (88.3)	66 (83.5)	0.25
Yes	51 (12.6)	38 (11.7)	13 (16.5)	
**Drinking history**				
No	318 (78.7)	259 (79.7)	59 (74.7)	0.33
Yes	86 (21.3)	66 (20.3)	20 (25.3)	
**History of falls**				
No	338 (83.7)	310 (95.4)	28 (35.4)	**0.007**
Yes	66 (16.3)	15 (4.6)	51 (64.6)	
**Comorbidity**				
No	201 (49.8)	178 (54.8)	23 (29.1)	**<0.001**
Yes	203 (50.2)	147 (45.2)	56 (70.9)	
**Polypharmacy**				
No	172 (42.6)	140 (43.1)	32 (40.5)	0.68
Yes	232 (57.4)	185 (56.9)	47 (59.5)	
**Physical activity**				
No	54 (13.4)	39 (12.0)	15 (19.0)	0.10
Yes	350 (86.6)	286 (88.0)	64 (81.0)	
**Participation in leisure activity**				
No	17 (4.2)	9 (2.8)	8 (10.1)	**0.003**
Yes	387 (95.8)	316 (97.2)	71 (89.9)	
**Participation in social activity**				
No	129 (31.9)	102 (31.4)	27 (34.2)	0.63
Yes	275 (68.1)	223 (68.6)	52 (65.8)	
**Sleep quality**				
Normal^b^	46 (11.4)	39 (12.0)	7 (8.9)	0.43
Poor^c^	358 (88.6)	286 (88.0)	72 (91.1)	

* Data are presented as number (percent).

a Chi-square test.

b Normal sleep quality indicates PSQI ≤ 7.

c Poor sleep quality indicates PSQI >7.

BMI, Body Mass Index.

Bold values represent statistically significant differences between comparison groups (*p*-value < 0.05 or 95% CI of OR not containing 1).

### 3.2 The relationship between sleep quality and fall risk among the study subjects

The results of the multivariate regression model presented in Table 2 show that religion, education level (elementary school or below), comorbidities, regular participation in recreational activities, and the average sleep quality score according to the PSQI are factors related to the fall risk among the elderly participants in the study.

**Table 2:** Multiple logistic regression analysis of factors related to fall risk.

**Variables**	***b***	**SE**	**Wald**	***P-*value**	**OR (95% CI)**
**Religion**					
No	Ref				
Yes	1.28	0.65	3.94	0.04	**3.60 (1.02, 12.77)**
**Education**					
College and above	Ref				
Junior school	1.27	0.85	2.23	0.14	3.55 (0.67, 18.69)
High school	1.10	0.74	2.22	0.14	3.00 (0.71, 12.78)
Primary school and below	1.94	0.76	6.48	0.01	**6.98 (1.56, 31.16)**
**History of falls**					
No	Ref				
Yes	2.14	0.24	79.34	< 0.001	**8.46 (5.29, 15.54)**
**Comorbidity**					
No	Ref				
Yes	1.55	0.42	13.46	< 0.001	**4.69 (2.05, 10.70)**
**Participate in leisure activity**					
No	Ref				
Yes	−1.99	0.70	8.09	0.004	**0.14 (0.04, 0.54)**
PSQI score	0.15	0.07	4.75	0.03	**1.16 (1.02, 1.32)**
Constant	−5.13	1.59	10.38	0.001	

SE, standard error; OR, odds ratio; CI, confidence interval; PSQI, Pittsburgh Sleep Quality Index. Bold values represent statistically significant differences between comparison groups (*p*-value < 0.05 or 95% CI of OR not containing 1).

In our primary analysis, poorer sleep quality (increased PSQI scores) was significantly associated with a higher risk of falls across all four models, as shown in Table 3. In the multivariate adjusted model, the adjusted OR was 1.16 (95% CI = 1.02, 1.32) with an increase in the overall PSQI score. In our secondary analysis, when the overall PSQI score was categorized into quartiles, the highest PSQI score quartile was significantly associated with an increased risk of falls compared to the lowest PSQI score quartile across all four models (Table 3). In the multivariate adjusted model, participants in the highest PSQI score quartile showed a higher fall rate compared to those in the lowest PSQI score quartile (Model 2, OR = 2.43; 95% CI = 1.00, 5.93), and there was a trend toward an increased fall rate compared to those in the lowest PSQI score quartile (Model 3, OR = 2.53; 95% CI = 0.93, 6.88).

**Table 3:** Logistic regression analysis of PSQI Score and risk of fall risk.

**Variable, unit/category**	**Fall risk**	**Unadjusted**	**Model 1^a^**	**Model 2^b^**	**Model 3^c^**
	***N*** = **404**	***n*** = **404**	***n*** = **404**	***n*** = **404**	***n*** = **404**
	***n*** **(%)**	**OR (95% CI)**	**OR (95% CI)**	**OR (95% CI)**	**OR (95% CI)**
Continuous variable^d^	11.76 ± 3.33	**1.14 (1.04, 1.25)**	**1.11 (1.01, 1.22)**	**1.18 (1.04. 1.33)**	**1.16 (1.02, 1.32)**
**Tertile**					
PSQI = 0–10 (*n* = 163)	32 (19.6)	1 (reference)	1 (reference)	1 (reference)	1 (reference)
PSQI = 10–12 (*n* = 149)	16 (10.7)	**0.49 (0.26, 0.94)**	**0.44 (0.23, 0.85)**	0.72 (0.31, 1.66)	0.65 (0.26, 1.64)
PSQI = 12–21 (*n* = 92)	31 (33.7)	**2.08 (1.17, 3.72)**	1.71 (0.93, 3.12)	2.17 (1.00, 4.82)	2.23 (0.93, 5.37)
**Quartiles**					
PSQI = 0–9 (*n* = 106)	20 (18.9)	1 (reference)	1 (reference)	1 (reference)	1 (reference)
PSQI = 9–11 (*n* = 134)	19 (14.2)	0.71 (0.36, 1.41)	0.70 (0.35, 1.40)	0.91 (0.37, 2.23)	0.79 (0.29, 2.16)
PSQI = 11–12 (*n* = 72)	9 (12.5)	0.61 (0.26, 1.44)	0.57 (0.24, 1.35)	1.00 (0.33, 2.98)	0.93 (0.28, 3.13)
PSQI = 12–21 (*n* = 92)	31 (33.7)	**2.19 (1.14, 4.19)**	1.86 (0.95, 3.64)	2.43 (1.00, 5.93)	2.53 (0.93, 6.88)

OR, odds ratio; CI, confidence interval; PSQI, Pittsburgh Sleep Quality Index.

a Model 1 adjusted for age and gender.

b Model 2 adjusted for age, gender, and any falls in the previous year.

c Model 3 adjusted for age, gender, any falls in the previous year, education level, smoking history, drinking history, participation in leisure activity, and comorbidity.

d Mean ± standard deviation (SD).

Bold values represent statistically significant differences between comparison groups (*p*-value < 0.05 or 95% CI of OR not containing 1).

## 4 Discussion

Our study was conducted to determine the correlation between fall risk and sleep quality among 404 elderly outpatients at Thai Binh University Hospital. The results showed that 79 elderly individuals were at risk of falls (accounting for 19.5%), while 325 elderly individuals were not at risk of falls. The differences in age, religion, education level, regular participation in recreational activities, and previous fall history between the two groups of elderly individuals with and without fall risk were statistically significant (*p* < 0.05). These findings are consistent with the results of Nguyen et al. (2022). Structural and functional changes in organ systems caused by medical conditions can directly affect posture control, balance, and gait in the elderly, thereby increasing the risk of falls. Moreover, during the investigation, the research team found that elderly individuals with a history of falls believed they were more prone to falling due to both physiological and psychological changes.

The results of the multivariate regression model presented show that elderly individuals who were religious, had an education level of elementary school or below, had a history of falls, had comorbidities, regularly participated in recreational activities, and had an average sleep quality score according to the PSQI were at higher risk of falls. Low education level has been identified as a risk factor for poor sleep quality in previous studies (Wu et al., 2018; Than et al., 2023; Tesfaye et al., 2024). According to the study by Wu et al. (2018), higher education has been shown to be a protective factor against poor sleep quality. This is because education is directly related to personal income and lifestyle factors, which significantly contribute to sleep quality.

Our study found that older adults who belong to a religion have a higher risk of falls than those who do not follow any religion. This finding is in contrast to a recent systematic review, which suggested that religious participation can have a positive impact on physical health (Kruk and Aboul-Enein, 2024). This result may be due to the limitations of convenient sampling method and a small sample size, because the study tended to interview a higher proportion of religious subjects with a history of falls than non-religious people. Moreover, in religious activities in Vietnam, where people, including the elderly, engage in a significant amount of movement, particularly climbing mountains, visiting temples and pagodas, as well as standing or sitting for long periods, which may increase the risk of falls. Further research is needed to determine the exact causal relationship between this factor and fall risk in older adults.

Multimorbidity has also been shown to be closely associated with poor sleep quality (Fu et al., 2020). The mechanism behind this correlation lies in the fact that individuals with multiple comorbidities often experience chronic pain, a major factor affecting sleep quality, especially at night. Our study found that poor sleep quality was a factor related to fall risk in the elderly, with adjusted odds ratio (OR) of 1.16 (95% CI = 1.02, 1.32) and a trend of increasing fall risk with higher overall PSQI scores. In secondary analysis, when overall PSQI scores were categorized into quartiles, the highest overall PSQI quartile was significantly associated with an increased risk of falls compared to the lowest PSQI quartile across all four models. This relationship has also been reported in previous studies (Lee et al., 2021; St George et al., 2009). These studies primarily focused on the relationship between individual aspects of sleep quality, particularly sleep duration, and found that short sleep duration may increase the risk of falls and injuries in the elderly. However, a meta-analysis of epidemiological studies showed that not only short sleep duration but also excessively long sleep duration may lead to a higher risk of falls in the elderly (Wu and Sun, 2017). The inconsistency in results across studies is partly due to differences in the confounding factors selected for analysis, such as socioeconomic variables, mental disorders, or the use of sleeping pills.

This study adds further evidence to the relationship between sleep quality and fall risk through a well-designed survey process on a large sample. Sleep variables, including sleep duration and sleep disorders, have been encouraged to be considered in studies related to sleep quality because these variables can affect study outcomes. Therefore, a notable strength of the current study is the simultaneous consideration of the relationship between sleep duration and sleep quality with fall risk in the elderly.

Our study has some limitations. The use of a cross-sectional design does not allow for causal inferences about the relationship between sleep quality and fall risk in the participants. Moreover, due to the time-varying nature of both sleep quality and fall risk variables, the study design used does not allow for determining which factor occurs first. Additionally, some factors related to injuries and mental disorders, which have been reported to be associated with falls, were not accounted for in the current study because no such data were collected. The information collected from the participants was based on self-report, so this study cannot exclude the possibility of recall bias. Another limitation is that the study was conducted at only one provincial hospital in a northern province of Vietnam, so the findings may not be representative of the broader Vietnamese population. Therefore, further cross-sectional and longitudinal studies in multiple centers are needed to provide comprehensive evidence on the factors affecting fall risk among Vietnamese elderly.

## 5 Conclusion

The results of the current study show a strong and significant relationship between sleep quality and fall risk among the elderly in a province in Northern Vietnam. Our research findings provide positive contributions to the health of the elderly community by suggesting that improving sleep quality may reduce falls and fall-related injuries among the elderly. Future prospective longitudinal studies are needed to investigate the causal relationship between sleep duration, sleep quality, and fall risk in the elderly.

## Data Availability

The datasets underlying the results of this study are available from the corresponding author upon reasonable request.
